# Use of Updated COVID-19 Vaccines 2023–2024 Formula for Persons Aged ≥6 Months: Recommendations of the Advisory Committee on Immunization Practices — United States, September 2023

**DOI:** 10.15585/mmwr.mm7242e1

**Published:** 2023-10-20

**Authors:** Joanna J. Regan, Danielle L. Moulia, Ruth Link-Gelles, Monica Godfrey, Josephine Mak, Morgan Najdowski, Hannah G. Rosenblum, Melisa M. Shah, Evelyn Twentyman, Sarah Meyer, Georgina Peacock, Natalie Thornburg, Fiona P. Havers, Sharon Saydah, Oliver Brooks, H. Keipp Talbot, Grace M. Lee, Beth P. Bell, Barbara E. Mahon, Matthew F. Daley, Katherine E. Fleming-Dutra, Megan Wallace

**Affiliations:** ^1^National Center for Immunization and Respiratory Diseases, CDC; ^2^Eagle Health Analytics, San Antonio, Texas; ^3^Watts Healthcare Corporation, Los Angeles, California; ^4^Vanderbilt University School of Medicine, Nashville, Tennessee; ^5^Stanford University School of Medicine, Stanford, California; ^6^University of Washington, Seattle, Washington; ^7^Institute for Health Research, Kaiser Permanente Colorado, Denver, Colorado,

**Summary**

**What is already known about this topic?**

Since September 2022, bivalent mRNA COVID-19 vaccines have been recommended in the United States, but the variants these vaccines were designed to protect against are no longer circulating widely. In September and October 2023, the Food and Drug Administration approved and authorized updated 2023–2024 Formula monovalent XBB.1.5 component–containing COVID-19 vaccines, formulated to target current variants more closely, specifically Omicron variant XBB.1.5, for persons aged ≥6 months.

**What is added by this report?**

On September 12, 2023, the Advisory Committee on Immunization Practices recommended vaccination with updated COVID-19 vaccines for all persons aged ≥6 months.

**What are the implications for public health practice?**

The updated COVID-19 vaccines are meant to broaden vaccine-induced immunity and provide protection against the currently circulating SARS-CoV-2 XBB-sublineage variants including against severe COVID-19–associated illness and death.

## Abstract

COVID-19 vaccines protect against severe COVID-19–associated outcomes, including hospitalization and death. As SARS-CoV-2 has evolved, and waning vaccine effectiveness has been noted, vaccine formulations and policies have been updated to provide continued protection against severe illness and death from COVID-19. Since September 2022, bivalent mRNA COVID-19 vaccines have been recommended in the United States, but the variants these vaccines protect against are no longer circulating widely. On September 11, 2023, the Food and Drug Administration (FDA) approved the updated (2023–2024 Formula) COVID-19 mRNA vaccines by Moderna and Pfizer-BioNTech for persons aged ≥12 years and authorized these vaccines for persons aged 6 months–11 years under Emergency Use Authorization (EUA). On October 3, 2023, FDA authorized the updated COVID-19 vaccine by Novavax for use in persons aged ≥12 years under EUA. The updated COVID-19 vaccines include a monovalent XBB.1.5 component, which is meant to broaden vaccine-induced immunity and provide protection against currently circulating SARS-CoV-2 XBB-sublineage variants including against severe COVID-19–associated illness and death. On September 12, 2023, the Advisory Committee on Immunization Practices recommended vaccination with updated COVID-19 vaccines for all persons aged ≥6 months. These recommendations will be reviewed as new evidence becomes available or new vaccines are approved and might be updated.

## Introduction

By the end of 2022, COVID-19 vaccines had prevented 18.5 million COVID-19 hospitalizations and 3.2 million COVID-19 deaths in the United States (*1*). As SARS-CoV-2 has evolved, and waning vaccine effectiveness (VE) has been observed, vaccine formulations and policies have been updated to provide continued protection against severe COVID-19–associated illness and death. On September 11, 2023, the Food and Drug Administration (FDA) authorized the updated (2023–2024 Formula) COVID-19 mRNA vaccines by Moderna and Pfizer-BioNTech for use in persons aged 6 months–11 years under Emergency Use Authorization (EUA) and approved the updated Moderna and Pfizer-BioNTech COVID-19 vaccines for persons aged ≥12 years (*2*). On October 3, 2023, FDA authorized the updated Novavax COVID-19 vaccine for use in persons aged ≥12 years under EUA (*2*). The updated COVID-19 vaccines include a monovalent XBB.1.5 component and are meant to broaden vaccine-induced immunity and provide increased protection (compared with protection from earlier vaccines that might have waned) against currently circulating SARS-CoV-2 XBB-sublineage variants, which, by September 2, 2023, accounted for >99% of sequenced SARS-CoV-2 specimens in the United States.* As of September 11, 2023, bivalent mRNA COVID-19 vaccines (based on the ancestral SARS-CoV-2 strain and BA.4/BA.5 variants) are no longer authorized for use in the United States, and as of October 3, 2023, original monovalent Novavax COVID-19 vaccines (based on the ancestral SARS-CoV-2 strain) are no longer authorized for use in the United States. On September 12, 2023, the Advisory Committee on Immunization Practices (ACIP) recommended vaccination with the updated COVID-19 vaccine for all persons aged ≥6 months. These recommendations will be reviewed as new evidence becomes available or new vaccines are approved and might be updated.

## Background

Although severe COVID-19 is now less prevalent in the United States than during previous years, it continues to cause significant morbidity and mortality in this country. Currently, older adults (aged ≥65 years) and infants aged <6 months are at highest risk for COVID-19–associated hospitalization. During January 1–August 26, 2023, COVID-19–associated hospitalization rates among adults aged ≥75 years were two to three times as high as those among the next youngest age group (adults aged 65–74 years). Rates among infants aged <6 months are similar to those among adults aged 65–74 years (*3*).

Nevertheless, persons aged 6 months–64 years, including those with no underlying medical conditions, remain at risk for severe COVID-19. Rates of COVID-19–associated hospitalization are currently lowest among children and adolescents aged 5–17 years. However, among persons in this age group who were hospitalized with COVID-19 during January–June 2023, 23% of those aged 5–11 years and 34% of those aged 12–17 years had no underlying medical conditions. During January 2022–June 2023, among children and adolescents aged ≤17 years who died during a COVID-19 hospitalization, 50% had no underlying condition. During January 1–July 22, 2023, a total of 28,140 persons, including 26 aged <1 year, 18 aged 1–4 years, 36 aged 5–19 years, 463 aged 15–44 years, 2,821 aged 45–64 years, and 24,776 aged ≥65 years, died from COVID-19, as evidenced by COVID-19 being listed as the underlying cause of death on the death certificate.^†^

Post–COVID-19 conditions contribute to COVID-19–related morbidity among all age groups. The prevalence of ongoing symptoms ≥3 months after COVID-19 illness ranged from <1% among persons aged <18 years to 5% among those aged 35–49 years. During June 7–19, 2023, approximately one in four adults with post–COVID-19 conditions reported significant activity limitations (*4*).

Members of racial and ethnic minority groups continue to be disproportionately affected by COVID-19–associated hospitalization (*5*). Higher prevalences of underlying conditions in some racial and ethnic minority populations might increase their risk for severe COVID-19–associated outcomes (*6*). As of May 10, 2023, only 17% of the U.S. population had received a bivalent COVID-19 vaccine dose, with lower coverage among some racial and ethnic minority populations, potentially driven by differences in vaccine access and acceptability (*5*,*7*).

After declining throughout the spring and early summer of 2023, COVID-19–associated hospitalization rates began increasing in mid-July 2023. Further increases are anticipated during the fall and winter respiratory virus season (*5*).

## Methods

Since June 2020, ACIP has convened 37 public meetings to review data relevant to the potential use of COVID-19 vaccines.^§^ The ACIP COVID-19 Vaccine Work Group, comprising experts in adult and pediatric medicine, obstetrics and gynecology, infectious diseases, vaccinology, vaccine safety, public health, and ethics, has met weekly to review COVID-19 surveillance data; evidence regarding immunogenicity, efficacy, effectiveness, and safety of COVID-19 vaccines; and implementation considerations. The Work Group conducted a systematic review of benefits and harms of vaccination, and used the Grading of Recommendations, Assessment, Development, and Evaluation (GRADE) methodology^¶^ to assess the certainty of the evidence regarding benefits and harms associated with a bivalent vaccine administered in the United States during September 2022–April 2023. The Work Group selected this population, intervention, and pandemic period of high seroprevalence to identify evidence most applicable to what can be anticipated from this year’s vaccine in the United States. The certainty of evidence was assessed separately for infants and children aged 6 months–11 years, and adolescents and adults aged ≥12 years based on the difference in recommended vaccine dosage for these two age groups. The Work Group also reviewed additional CDC data on VE and safety, as well as data on the updated vaccines provided by manufacturers (*8*–*10*). To assess the evidence for benefits and harms associated with COVID-19 vaccine use, and to guide deliberations, ACIP uses the Evidence to Recommendations (EtR) Framework.** Within this framework, ACIP considered the importance of COVID-19 as a public health problem, including during the Omicron XBB-lineage–predominant era (January 2023–September 2023), as well as issues of resource use, benefits and harms, patients’ values, acceptability, feasibility, and equity related to vaccine use. ACIP evaluated data related to all vaccines for which updated 2023–2024 formulations were anticipated (i.e., Moderna, Novavax, and Pfizer-BioNTech).

## Vaccine Effectiveness and Safety

Published assessments of previous vaccine formulations’ VE and safety were evaluated using GRADE. GRADE is used to assess the confidence (high, moderate, low, or very low) that the true effect lies close to that of the estimated effect. Evidence that includes only randomized controlled trials begins at high certainty, whereas evidence that includes observational data begins at low certainty.

Among adolescents and adults, benefits of bivalent vaccination were assessed using pooled observational VE data for three outcomes: medically attended COVID-19,^††^ hospitalization attributed to COVID-19, and death attributed to COVID-19. Pooled VE against medically attended COVID-19 was 53% (95% CI = 50%–56%), and hospitalization attributed to COVID-19 was 48% (95% CI = 30%–61%). For both critical outcomes, the certainty assessment was low.^§§^ Pooled VE against death attributed to COVID-19 was 61% (95% CI = 41%–74%), and the certainty assessment was very low because of serious concern for inconsistency. Among infants and children, insufficient observational data were identified for a systematic review of benefits, but benefits were indirectly inferred from adolescent and adult data. The certainty assessment was very low for all three outcomes because of serious concern for indirectness.

Studies from the Vaccine Safety Datalink (VSD), a postauthorization vaccine safety monitoring system, were used to assess rates of serious adverse events (i.e., myocarditis or pericarditis and anaphylaxis, which were the outcomes specified for GRADE) that have been associated with vaccination (myocarditis after receipt of COVID-19 vaccine has been reported primarily in adolescent and young adult males)^¶¶^ (*11*), and the certainty assessment was low among adolescents and adults and very low among infants and children. Severe reactogenicity (grade ≥3*** local or systemic reactions) was assessed using pooled clinical trial data after any original monovalent primary series dose. Severe reactogenicity occurred more often in the vaccine than placebo study arms, and the certainty assessment for the clinical trial body of evidence was low because of very serious concern for indirectness^†††^ in both age groups. The GRADE evidence profile is available at www.cdc.gov/vaccines/acip/recs/grade/covid-19-2023-2024-Monovalent.html.

Additional, updated CDC VE data were also reviewed, including data showing patterns of waning bivalent vaccine–induced immunity against infection and COVID-19–associated hospitalization during a period with increased Omicron XBB sublineage circulation (*12*,*13*). During September 2022–August 2023, VE against hospitalization among adults aged ≥65 years without an immunocompromising condition waned from 67% (95% CI = 62%–71%) at 7–59 days postvaccination to 28% (95% CI = 18%–36%) at 60–119 days (*13*). VE of both the original monovalent and bivalent vaccines against critical outcomes (invasive mechanical ventilation, intensive care unit admission, or death) has remained more durable than VE against less severe outcomes among adults, including those with and without immunocompromising conditions (*12*,*14*). VE patterns were similar among children and adults, although available data were more limited in children (*13*,*15*). VE against emergency department and urgent care visits among persons aged 5–17 years ranged from 59%–63% by age group 7–59 days after a bivalent dose, waning to 36%–47% by age group 60–119 days after a bivalent dose (*13*). VE has historically been lower and has waned more quickly among adults with immunocompromise than among immunocompetent adults, although bivalent VE trends are less clear (*12*,*13*).

Additional, updated data on COVID-19 vaccine safety from VSD were also reviewed. The risk for myocarditis or pericarditis after receipt of a bivalent vaccine dose is uncertain because myocarditis is a rare outcome, and bivalent vaccination coverage is relatively low, especially in adolescents and young adults. Myocarditis rates after booster doses in adolescent and young adult males are lower than rates after primary series vaccination, but estimates for monovalent booster and bivalent doses are limited by the lower numbers of doses administered in VSD in this group (*16*). A longer interval between doses has been associated with lower rates of myocarditis (*17*).

ACIP recommendations for the updated COVID-19 vaccines were also guided by data on immunogenicity provided by the vaccine manufacturers. Data from Moderna, Novavax, and Pfizer-BioNTech show that monovalent XBB component–containing COVID-19 vaccines increase the immune response against the currently circulating XBB-sublineage variants (*8*–*10*). The evidence used to guide EtR is available at https://www.cdc.gov/vaccines/acip/recs/grade/covid-19-2023-2024-Monovalent-etr.html.

## Cost Effectiveness

COVID-19 vaccination is a cost-effective intervention, particularly in adults aged ≥65 years, among whom incidence is highest. For this age group, a dose of the vaccine is cost saving (at an assumed cost of $120 per dose). Among adults aged 50–64 years, the incremental cost-effectiveness ratio of updated COVID-19 vaccines was estimated to be $25,787 per quality-adjusted life year, with estimates in those aged ≥50 years robust to input changes across plausible ranges (*18*). For adults aged 18–49 years, the incremental cost-effectiveness ratio for updated COVID-19 vaccines was estimated to be $115,588 per quality-adjusted life year, although estimates in younger adults were more sensitive to changes in input, with higher VE or hospitalization rates increasing cost-effectiveness (*18*). Cost-effectiveness estimates are not yet available for pediatric populations (*18*).

## Recommendations for Use of 2023–2024 COVID-19 Vaccines in Persons Aged ≥6 Months

On September 12, 2023, ACIP recommended vaccination with the updated (2023–2024 Formula) COVID-19 vaccine for all persons aged ≥6 months.^§§§^ The recommendation is inclusive of FDA-licensed or authorized updated monovalent XBB component–containing COVID-19 vaccines (i.e., Moderna, Novavax and Pfizer-BioNTech updated COVID-19 vaccines), consistent with the FDA-licensed indication or EUA. The recommendation for children aged 6 months–11 years is an interim recommendation because the updated COVID-19 vaccines for this age group are currently authorized under EUA. In addition, the recommendation for the updated Novavax COVID-19 vaccine is an interim recommendation because the Novavax COVID-19 vaccine is currently authorized under EUA. 

Infants and children aged 6 months–4 years are recommended to receive a multidose initial series (previously referred to as the primary series) and at least 1 updated mRNA COVID-19 vaccine dose depending on vaccination history as defined herein. Infants and children aged 6 months–4 years who are unvaccinated are recommended to receive either 2 updated Moderna COVID-19 vaccine doses or 3 updated Pfizer-BioNTech COVID-19 vaccine doses (Table 1). Infants and children aged 6 months–4 years who previously received original monovalent or bivalent mRNA vaccine doses are recommended to receive 1 or 2 homologous (i.e., from the same manufacturer) updated COVID-19 mRNA vaccine doses, depending on vaccine manufacturer and the number of previous vaccine doses received. Infants and children aged 6 months–4 years who completed the initial series with original monovalent or bivalent mRNA vaccine doses are recommended to receive 1 updated COVID-19 vaccine dose, at least 2 months after receipt of the last COVID-19 vaccine dose. Infants and children aged 6 months–4 years may receive either the updated Moderna or Pfizer-BioNTech COVID-19 vaccine; however, all doses administered to an infant or child in this age group should be from the same manufacturer.

**TABLE 1:** Recommended COVID-19 vaccination schedule for persons aged 6 months–4 years who are not moderately or severely immunocompromised,* by COVID-19 vaccination history — United States, September 2023

Previous COVID-19 vaccination history (before updated mRNA vaccine)^†^	Updated mRNA vaccine	No. of updated mRNA vaccine doses indicated	Interval between doses
**Unvaccinated**	Moderna	2	Dose 1 and dose 2: 4–8 wks
	Pfizer-BioNTech	3	Dose 1 and dose 2: 3–8 wks Dose 2 and dose 3: ≥8 wks
**Received Moderna vaccine**			
1 dose any Moderna	Moderna	1	4–8 wks after last dose
≥2 doses any Moderna	Moderna	1	≥8 wks after last dose
**Received Pfizer-BioNTech vaccine**			
1 dose any Pfizer-BioNTech	Pfizer-BioNTech	2	Dose 1: 3–8 wks after last dose Dose 1 and dose 2: ≥8 wks
2 doses any Pfizer-BioNTech	Pfizer-BioNTech	1	≥8 wks after last dose
≥3 doses any Pfizer-BioNTech	Pfizer-BioNTech	1	≥8 wks after last dose

* Additional clinical considerations, including detailed schedules and tables by age and vaccination history for those who are and are not moderately or severely immunocompromised, are available. https://www.cdc.gov/vaccines/covid-19/clinical-considerations/covid-19-vaccines-us.html

^†^ https://www.cdc.gov/vaccines/covid-19/clinical-considerations/interim-considerations-us.html#not-immunocompromised

For those receiving updated mRNA COVID-19 vaccines, persons aged ≥5 years without immunocompromise are recommended to receive 1 updated COVID-19 vaccine dose, irrespective of previous COVID-19 vaccination history (Table 2). For those receiving updated Novavax COVID-19 vaccines, persons ages ≥12 years without immunocompromise are recommended to receive 2 updated COVID-19 vaccine doses if previously unvaccinated and 1 updated dose if previously vaccinated with any COVID-19 vaccine. For those who have received previous COVID-19 vaccines, the updated vaccine should be administered ≥2 months after receipt of the most recent dose.

**TABLE 2:** Recommended COVID-19 vaccination schedule for persons aged ≥5 years who are not moderately or severely immunocompromised,* by COVID-19 vaccination history — United States, September 2023

COVID-19 vaccination history before updated vaccine^†^	Updated vaccine	No. of updated doses indicated	Interval between doses
Unvaccinated	Moderna	1	—
	Pfizer-BioNTech	1	—
	Novavax (aged ≥12 yrs only)	2	Dose 1 and dose 2: 3–8 wks
Receipt of ≥1 COVID-19 vaccine dose, including Moderna, Pfizer-BioNTech, Novavax (aged ≥12 yrs only), or Janssen (Johnson & Johnson) (aged ≥18 yrs only)	Moderna	1	≥8 wks after last dose
	Pfizer-BioNTech	1	≥8 wks after last dose
	Novavax (aged ≥12 yrs only)	1	≥8 wks after last dose

* Additional clinical considerations, including detailed schedules and tables by age and vaccination history for those who are and are not moderately or severely immunocompromised, are available. https://www.cdc.gov/vaccines/covid-19/clinical-considerations/covid-19-vaccines-us.html

^†^ https://www.cdc.gov/vaccines/covid-19/clinical-considerations/interim-considerations-us.html#not-immunocompromised

## Recommendations for 2023–2024 COVID-19 Vaccines in Persons Aged ≥6 Months Who Are Moderately or Severely Immunocompromised

Unvaccinated persons aged 6 months–11 years who are moderately or severely immunocompromised are recommended to receive an initial vaccination series of 3 homologous updated (2023–2024 Formula) mRNA COVID-19 vaccine doses. Unvaccinated persons aged ≥12 years who are moderately or severely immunocompromised can complete an initial vaccination series with 3 homologous doses of updated mRNA or 2 doses of updated Novavax COVID-19 vaccine.^¶¶¶^ Persons aged ≥6 months who are moderately or severely immunocompromised and previously received 1 or 2 original monovalent or bivalent mRNA vaccine doses are recommended to receive 1 or 2 homologous updated COVID-19 vaccine doses, depending on the number of previous vaccine doses. Persons aged ≥6 months who are moderately or severely immunocompromised who previously received ≥3 original monovalent or bivalent mRNA vaccine doses are recommended to receive 1 updated COVID-19 vaccine dose. Persons aged ≥12 years who are moderately or severely immunocompromised and who previously received original Novavax COVID-19 vaccine or Janssen (Johnson & Johnson) COVID-19 vaccine, including those who also received original monovalent or bivalent mRNA COVID-19 vaccine doses, are recommended to receive 1 updated COVID-19 vaccine dose from any FDA-authorized or approved manufacturer.

Persons who are moderately or severely immunocompromised, have completed an initial series, and have received ≥1 updated COVID-19 vaccine dose, may receive additional updated COVID-19 vaccine doses, guided by the clinical judgment of a health care provider and personal preference and circumstances. Any further additional doses should be administered ≥2 months after the last COVID-19 vaccine dose. Additional clinical considerations, including detailed schedules and tables by age and vaccination history for those who are and are not moderately or severely immunocompromised, are available at https://www.cdc.gov/vaccines/covid-19/clinical-considerations/covid-19-vaccines-us.html.

## Implementation Considerations

COVID-19 vaccines are transitioning from federal procurement and distribution into the commercial marketplace during fall 2023. Under the Affordable Care Act (ACA), ACIP recommendations for routine immunization that have been adopted by CDC and are listed on CDC Immunization Schedules are required to be covered by group health plans and health insurance issuers offering group or individual health insurance coverage without cost-sharing requirements. The Coronavirus Aid, Relief, and Economic Security (CARES) Act expedited coverage for COVID-19 vaccines; since January 5, 2021, ACA-covered insurers must cover, without cost sharing, any COVID-19 vaccine FDA authorized under an EUA or FDA approved under a Biologics License Application immediately upon authorization or approval of the vaccine (*19*). Thus, for U.S. residents with applicable ACA commercial medical insurance coverage, COVID-19 vaccines will be covered immediately. In addition, COVID-19 vaccines are covered under Medicare Part B, and nearly all Medicaid beneficiaries can receive COVID-19 vaccines without cost-sharing. COVID-19 vaccines are also included in the Vaccines for Children Program,**** which provides vaccines to approximately one half of U.S. persons aged <19 years at no cost. The Bridge Access Program for COVID-19 Vaccines is a public-private partnership serving as a temporary measure to maintain access to COVID-19 vaccines for adults who are uninsured or underinsured, working through both public health clinics and participating retail pharmacies.^††††^ Before vaccination, providers should provide the EUA Fact Sheet,^§§§§^ manufacturer’s package insert, or other written materials regarding the vaccine being administered and counsel vaccine recipients about expected systemic and local adverse reactions (reactogenicity).

## Reporting of Vaccine Adverse Events

Adverse events after vaccination should be reported to the Vaccine Adverse Event Reporting System (VAERS). Reporting is encouraged for any clinically significant adverse event even if it is uncertain whether the vaccine caused the event. Information on how to submit a report to VAERS is available at https://vaers.hhs.gov or by telephone at 1-800-822-7967.

## Footnotes

*
https://covid.cdc.gov/covid-data-tracker/#variant-proportions

† https://wonder.cdc.gov/mcd-icd10-provisional.html (Accessed September 7, 2023).

§ https://www.cdc.gov/vaccines/hcp/acip-recs/vacc-specific/covid-19.html (Accessed September 7, 2023).

¶
https://www.cdc.gov/vaccines/acip/recs/grade/about-grade.html

** https://www.cdc.gov/vaccines/acip/recs/grade/downloads/acip-evidence-recs-framework.pdf

†† Medically attended COVID-19 was defined as an emergency department or urgent care visit.

§§ Evidence that includes observational data starts at low certainty.

¶¶ Among persons aged ≥12 years, based on events occurring in a 0–1 day risk interval after either dose of primary series vaccination, the estimated incidence of confirmed anaphylaxis among adolescents and adults was 4.8 (95% CI = 3.2–6.9) per million doses of Pfizer-BioNTech COVID-19 vaccine and 5.1 (95% CI = 3.3–7.4) per million doses of Moderna COVID-19 vaccine. Among persons aged 12–39 years, based on events occurring in 7-day risk interval after vaccination versus a comparison interval in vaccinated persons, rates of chart-reviewed myocarditis or pericarditis per one million doses, were as high as 188 (95% CI = 86.0–356.9) in males aged 16–17 years after a monovalent booster dose of Pfizer-BioNTech COVID-19 vaccine.

*** Grade 3 or 4 reactogenicity is generally defined as reactions that prevent daily routine activity, require use of a pain reliever, or require an emergency department visit or hospitalization. Definitions used for each clinical trial are provided on CDC webpages. (https://www.cdc.gov/vaccines/covid-19/info-by-product/pfizer/reactogenicity.html; https://www.cdc.gov/vaccines/covid-19/info-by-product/moderna/reactogenicity.html)

††† Very serious concern for indirectness was noted because the body of evidence did not include anyone who received an updated dose, was from an earlier period of the pandemic, and excluded persons with previous COVID-19 infection, pregnant or breastfeeding women, and persons who were immunocompromised.

§§§ ACIP voted (13 to one) to recommend vaccination with 2023–2024 (monovalent, XBB-containing) COVID-19 vaccines as authorized under EUA or approved by Biologics License Application in persons aged ≥6 months.

¶¶¶ Apart from the administration of additional doses, the FDA EUA for Novavax COVID-19 vaccine does not provide for a specific vaccination schedule for persons who are moderately or severely immunocompromised.

****
https://www.cdc.gov/vaccines/programs/vfc/index.html

†††† https://www.cdc.gov/vaccines/programs/bridge/index.html (Accessed September 7, 2023).

§§§§
https://www.cdc.gov/vaccines/covid-19/eua/index.html
