# *Erratum:* Vol. 72, No. 42

**DOI:** 10.15585/mmwr.mm7249a6

**Published:** 2023-12-08

The report, “Use of Updated COVID-19 Vaccines 2023–2024 Formula for Persons Aged ≥6 Months: Recommendations of the Advisory Committee on Immunization Practices — United States, September 2023,” contained several errors.

On page 1141, the last sentence of the first complete paragraph should have read, “During January 1–July 22, 2023, a total of **28,128** persons, including 26 aged <1 year, 18 aged 1–4 years, 36 aged 5–19 years, **451** aged **20**–44 years, 2,821 aged 45–64 years, and 24,776 aged ≥65 years, died from COVID-19, as evidenced by COVID-19 being listed as the underlying cause of death on the death certificate.^†^”

On page 1142, the second sentence of the second complete paragraph should have read, “During September 2022–August 2023, VE against hospitalization among adults aged ≥65 years without an immunocompromising condition waned from 67% (95% CI = 62%–71%) at 7–59 days postvaccination to 28% (95% CI = 18%–36%) at **120–179** days (*13*).” In addition, the fifth sentence of the second complete paragraph should have read, “VE against emergency department and urgent care visits among persons aged 5–17**, 18–64, and ≥65** years ranged from 59%–63% by age group 7–59 days after a bivalent dose, waning to 36%–47% by age group 60–119 days after a bivalent dose (*13*).”

